# Good character: the implications of personality development and psychopathology for citizenship

**DOI:** 10.1192/bjb.2024.60

**Published:** 2025-04

**Authors:** Stephen Attard, Solange Valdez-Symonds, Steve Valdez-Symonds, Andrew Iles, Frances Maclennan

**Affiliations:** 1 Central and North West London NHS Foundation Trust, London, UK; 2 Project for the Registration of Children as British Citizens, London, UK; 3 Amnesty International UK, London, UK; 4 Surrey and Borders NHS Foundation Trust, Leatherhead, UK

**Keywords:** Childhood experience, conduct disorders, human rights, psychiatry and law, trauma and stressor-related disorders

## Abstract

The introduction of new legislation in 2006 brought about changes to the way citizenship applications were considered in the UK. Over the intervening years, several hundred children born in the UK have been denied British citizenship as a result of changes to the ‘good character’ requirement in the legislation – namely its extension to cover all those aged 10 years or older applying for citizenship, including individuals who were born in the UK. As a result of the formulaic way in which this requirement is assessed, citizenship can be denied on the basis of historical patterns of behaviour or offending from childhood. This article will consider whether the current approach to assessment of character in the context of applications for British citizenship is meaningful or appropriate, given developments in our understanding of normative psychological and neurological development and also the impact of psychosocial adversity, trauma, and broader psychopathological or neurodevelopmental conditions.

Since the introduction of the Immigration, Asylum and Nationality Act 2006, it is estimated that several hundred children born in the UK have been denied British citizenship. This is despite the fact that many of those children were raised in the UK, may never have left the country, identify as British, have their primary network of familial and social links here, and have statutory rights to citizenship. The Act, which brought in amendments to the British Nationality Act 1981, controversially extended the ‘good character’ requirement to all those aged 10 years or over at the time of their application for citizenship.^1^

When the British Nationality Act 1981 first took effect, it applied a good character requirement only for the naturalisation of adult migrants to the UK. The Act did not make the rights of children to register as British citizens subject to any good character requirement. A key concern behind the conception of this Act was that a child born in the UK but with no other connection to the UK, who had left at a very young age and never returned, should not be a British citizen or able to pass on British citizenship to their own children. However, there was recognition that children born and growing up in the UK should be British citizens by entitlement.

In December 2006, however, the good character requirement was extended to registration of British citizenship for children as well as adults, by section 58 of the Immigration, Asylum and Nationality Act 2006. The primary reason given was to bring registration in line with naturalisation, despite there having originally been a clear distinction made in the British Nationality Act 1981 for the above reasons. An inspection of the Home Office's application of the good character requirement in the case of young persons who apply for registration as British citizens carried out by the Independent Chief Inspector of Borders and Immigration in 2017 suggested that Home Office policy ‘had tightened in relation to the good character requirement [since December 2012], so that young persons were now subject to the same guidance as adults’.^2^

At present, the requirement is applied to people who have lived in the UK from their birth or after being brought here at a young age, in the same way it is applied to adults seeking to naturalise after migrating to the UK. Thus, children born in the UK and without any connection to another territory can be barred from citizenship and made subject to Home Office immigration powers, by a judgement of character. Such a judgement is made on the basis of several factors including dishonesty, ‘notoriety’, having an undischarged hospital order under the Mental Health Act, and a fixed formula concerning historical contact with the criminal justice system. If barred from British citizenship, these individuals could face being deported from the country in which they have been born or lived from an early age, to ‘home’ territories to which they may never have been or where they may have no family connections or social support network. A Freedom of Information application outlined in the *Guardian* newspaper in 2018 highlighted that 35 applications were rejected in 2017 and 59 in 2016, with a peak in the number of rejections at 78 in 2013.^3^

Perhaps understandably, concerns have been raised regarding the implementation of these powers in the cases of children. However, in 2019, the government's response to the seventh report from the Joint Committee on Human Rights put forward the position that ‘[t]he good character requirement applies to those aged 10 and over as that is the age of criminal responsibility. Children as young as 10 can and do commit very serious acts of criminality such as murder and rape, and the Government does not consider it appropriate to adjust the good character policy so that such acts would effectively become inadmissible when assessing a minor's suitability for British citizenship’.^4^

While acknowledging that specific critique of the current legislation would be beyond the scope of forensic psychiatry, in this article we touch on the interface between such legislation and psychiatry. We propose that formulating an opinion on aspects of an individual's character at such an age and applying a static formula to do so is fundamentally misconceived. The current process risks inappropriately depriving children of citizenship of the country in which they have been born and raised. Fundamental to the consideration of whether the assessment of a child's character offers a reliable assessment in relation to citizenship are concepts including personality, neurological development, and consideration of lifetime patterns of antisocial behaviour and desistance from crime.

## Current legislation

The Home Office document *Nationality: Good Character Requirement* (version 2)^1^ sets out the types of conduct which must be considered when assessing whether a person has satisfied the requirements of good character. However, it does not provide an exhaustive list of positive or negative factors. The factors to consider include an individual's financial soundness – for example, whether they have failed to pay taxes for which they were liable or have accrued significant debt. Dishonesty in relation to dealings with the British government, such as falsely claiming benefits, and a history of breaching immigration-related laws are also considered. Criminality and the ill-defined construct of ‘notoriety’ within the individual's local community are further factors to be considered. These factors are all based on markers of historical behaviour or potentially hearsay accounts; they do not directly measure or describe aspects of an individual's personality or character, suggesting that they are used as proxy measures for an individual's character.

With specific regard to the items on criminality, the guidance stipulates that citizenship should not be granted, save in exceptional circumstances, to a person who has been:

- sentenced to a period of imprisonment of 4 years or more; or
- sentenced to between 12 months and 4 years imprisonment, unless 15 years have passed since the end of the sentence; or
- sentenced to less than 12 months imprisonment, unless 10 years have passed since the end of the sentence; or
- convicted of a non-custodial offence or cautioned in the past 3 years (this can include the imposition of a fine or even a conviction resultant from non-payment of a fixed penalty notice); or
- subject to a hospital order or restricted hospital order that has not been fully discharged (the guidance stipulates refusal of the application irrespective of when the person was subject to the order).

Although mitigating facts relevant to the child's particular circumstances and best interests must be considered, the identified factors are considered in applicants from when they reach the age of 10 years. The behaviour and criminality of adult applicants during their childhood is also of significance, given the fixed formula applied to duration of custodial sentences outlined above.

Although we acknowledge that historical offending is an undeniable risk factor in relation to future such behaviour, the application of such a fixed formula for assessment of an individual's ‘character’ on the basis of juvenile offending, from such a young age, is simplistic and flawed. There is a low threshold identified above, for example, the inclusion of fines or convictions for non-payment of fixed penalty notices. It is also of particular concern that citizenship should not be granted in cases of a non-discharged hospital order or restricted hospital order, as this is suggestive of such individuals being at disadvantage as a result of their mental disorder.

## The construct of character

‘Character’ is not recognised medical terminology, and neither is it defined within the existing legislation of the British Nationality Act 1981. Whereas character and personality are not directly interchangeable in meaning, there is significant overlap between the two concepts, although character is more associated with an individual's ethical standards and principals. Indeed, the notion of character as a descriptor of the moral qualities and conduct of an individual held sway from the classical period to the late 19th century. Subsequently, texts including Ribot's *The Diseases of Personality*^5^ signalled a definitive shift toward the construct of personality, following the reconceptualisation and, to a degree, medicalisation of the term personality.

Into the early 20th century, the psychoanalytic model driven by Freud, with its focus on the unconscious mind and the role of early childhood experiences, led to a greater emphasis on understanding individual personalities rather than just moral character. Concepts such as the id, ego and superego were introduced, and consideration of the complexities of human personality structures, including internal conflicts, unresolved childhood issues and defence mechanisms, suggested that these are where criminal behaviours might be rooted. Such thoughts challenged more traditional character- or moral-based assumptions as explanations for criminality.^6^

As Melanie Klein expanded upon Freud's work by focusing on the early stages of psychological development and the formation of internalised object relations, she emphasised the significance of early relationships, particularly with primary caregivers, in shaping an individual's character structure.^7^ By the mid-century, Eysenck's proposed hierarchical model of personality, which included the dimensions of extraversion/introversion, neuroticism/emotional stability and psychoticism, began to influence thought on the links between personality and offending. For example, he suggested that individuals with certain personality traits, such as high levels of neuroticism and low levels of conscientiousness, may be more predisposed to engage in criminal behaviour.^8^

By highlighting the interplay between early experiences, unconscious processes and personality dynamics in shaping human behaviour, 20th-century psychoanalytic theory was influential in the development of current models in psychology and criminology. Social learning theory and Robert Hare's conceptualisation of psychopathy were, among others, important landmarks in understanding offending behaviour. Current psychological and medical literature remains focused on personality, trauma, cognitive function, psychopathology and broader social functioning when considering drivers behind offending behaviour. The fact that the term ‘character’ has long been conceptualised within a classical framework perhaps calls into question the anachronistic nature of its use within present-day legislation.

## Risks inherent in using the ‘good character’ requirement for all those aged 10 or over at the time of their application for citizenship

The potential risks of applying a good character test to children from the age of 10 are manifold. They include the dynamic nature of personality, as well as broader normative brain development, behavioural changes in adulthood, and the presence of mental disorder or neurodevelopmental conditions. The concept of personality structure encompasses the fundamental ‘operating system’ or underlying stable configuration of personality, which in turn manifests itself in specific, observable traits of personality functioning.^8^ It is on this basis that the dimensional approach to describing personality disorder within the ICD-11 was formulated.

Personality traits generally refer to an individual's tendency to behave, think and feel in relatively consistent ways across situations and time. They are essentially a collection of characteristics or traits, including the way we perceive and interpret the world, experience and manage our emotions, control our impulses or needs, and establish and maintain relationships with other people. They are characteristics we develop as we age, and which make each of us an individual. Thus, as noted above, in many ways they are broadly analogous to character. The dynamic nature of personality development is suggestive of there being inherent risks in basing judgements for citizenship applications on a formula for good character.

Through early relationships, children develop mental representations of who they are in relation to others and of the availability and responsiveness of others in times of stress and need. To a significant degree, early attachment experiences predict adult attachment. Experience of interference with the development of attachments between a child and their caregivers can be a contributory factor in the development of childhood problems with behaviour, ability to deal with emotions and trusting people, which can further precipitate the development of an adult personality disorder in vulnerable individuals.^9^

By school age, children's personality traits are structured much as those of adults. Both children and adults are considered to exhibit the ‘big five’ major personality traits: extraversion, neuroticism, conscientiousness, agreeableness, and openness to experience.^10^ Through adolescence and young adulthood, there is significant development and normative change in relation to an individual's personality traits. Neuroticism, for example, increases during adolescence and then decreases in young adulthood. Agreeableness and conscientiousness are at their lowest levels in adolescence and then increase in young adulthood and middle age.^11^ Correspondingly, many characteristic manifestations of personality disorder reach their peak in adolescence and early adulthood.^10,12^ Some behavioural manifestations of such traits, including antisocial behaviour, also peak during adolescence and later improve.^13^ These factors are significant when considering that the peak age of offending behaviour in males is late adolescence, with desistence commonly following into adulthood.^14^

With regard to personality pathology, although maladaptive personality traits can be identified throughout adolescence, it may be prudent to avoid making a definitive diagnosis of personality disorder before the age of 25 years, given the particularly dynamic nature of personality traits and the presence of other confounding factors, such as ongoing brain development, before that age. The evidence for this judicious approach to diagnosis is elaborated below.

There is substantial evidence in the literature highlighting the significant cognitive changes and neurological development that take place far beyond childhood. Cognitive control is not fully developed until adulthood, because the prefrontal cortex is limited in its development until that time. This brain region is involved in the regulation of emotions, impulse control and ability to judge consequences, as well as the capacity to exercise good judgement, self-regulation and planning, and it is still maturing into early adulthood.^13^ Indeed, this region of the brain begins an extended process of development during adolescence. During that time, the frontal lobe experiences the greatest and most important structural change in the brain, a process which is not complete until nearer the age of 25 years.^14^

The changes flowing from this development have been described as reflecting the ‘maturity principle’ of personality development. This states that during young adulthood, most individuals become more cautious and self-controlled and less prone to negative emotions.^12^ The maturational hypothesis is based broadly on adolescents typically becoming more emotionally stable, interpersonally more sophisticated and skilled, and intellectually more knowledgeable and future-oriented with age. These changes, in turn, increase moral reasoning, reduce impulsivity and facilitate more future-oriented goals and planning.

In addition to consideration of the above, there are a multitude of social, environmental and psychological factors which influence the risk of children, adolescents and young adults engaging in antisocial or offending behaviour. Such factors further influence desistence from offending across an individual's later life course. Research has shown that in general terms, desistance from offending as an individual reaches adulthood is common, and previous offending records have little predictive validity regarding lifelong patterns of offending.^15^

Although conduct disorder in childhood and adolescence is strongly associated with antisocial or offending behaviour at that time, it is of note that this is not invariably a life-course disorder. There are very significant differences between those considered to have a life-course-persistent form of the disorder and those with adolescence-limited conduct disorder, despite their patterns of antisocial behaviour appearing broadly similar during adolescence.^16^

Children with early-onset or life-course-persistent conduct disorder are significantly more likely to go on to develop a dissocial or emotionally unstable personality disorder in adulthood and continue to engage in antisocial or offending behaviour. This is in contrast to those with adolescence-limited conduct disorder, who are very much more likely to have better outcomes by mid-life, including desistence of offending or positive work and family life. The prevalence of these disorders is markedly different, with adolescence-limited conduct disorder being more than twice as common as life-course-persistent conduct disorder.^17^ Thus, applying a rigid formula to historical offending would be unlikely to reliably differentiate between these two groups, who could potentially have very different adult trajectories.

Aside from normative personality and cognitive changes, the presence of affective or psychotic illness, intellectual disability, brain injury, substance misuse disorder or neurodevelopmental conditions such as attention-deficit hyperactivity disorder (ADHD) or autistic spectrum conditions can be significant in relation to offending or antisocial behaviour in childhood and adolescence. Such factors, which are often identified during childhood or adolescence, are of course also amenable to change. Treatment interventions for mental disorders can have a profound impact on behaviour and offending. This can be seen, for example, in relation to successful treatment of ADHD. Pharmacological therapy with stimulant medication can substantially reduce an individual's degree of impulsivity and antisocial behaviour. Studies in Sweden, for example, have demonstrated a reduction of up to 30% in offending behaviour in those treated with stimulant medication.^18^

The literature demonstrates a clear link between trauma and antisocial behaviour, showing that children who experience abuse are at a greater risk of being arrested in adolescence.^19^ Within this context, trauma-informed psychological interventions can also reduce the risk of offending behaviour in this group.

## Conclusion

Given the complexity of the non-exhaustive factors highlighted above and the stakes at play for the individual, it is clear that a simplistic, formulaic approach to the assessment of character on the basis of a handful of static historical factors, including the length of sentence handed down to a young offender, is inadequate in relation to considering an individual's character or their future character as an adult. In the years since the legislation was enacted, hundreds of children have been denied citizenship based on this model, despite having been born in this country. We question its validity and highlight the probable detrimental impact the process has on the children who fall under its remit. It is possible that children denied citizenship in this manner may be sent to countries they have never visited, where they do not speak the principal language, and where they have no social network or support.

The changes enacted by section 58 of the Immigration, Asylum and Nationality Act 2006 run contrary to the spirit of the British Nationality Act 1981 as it was envisaged, for children born in the UK. The application of the good character ‘test’ to this group from the age of 10 is unlikely to be predictive of future risk or societal contribution; however, the potential consequences of being denied citizenship for the children affected are profound.

We argue that the extension of the good character requirement to all those applying for British citizenship from the age of 10 should be reviewed and reconsidered. While this legislation remains in place, at the least, any decisions taken by the Home Office in relation to children born in the UK applying for citizenship should be informed by detailed multi-agency assessments covering the breadth of social, psychological and medical factors which can influence future offending behaviour. Such assessments could further address whether and what strategies could be put in place to ameliorate the risks to the point that citizenship could be considered. To consider these factors, a decision-making panel could be convened, which could include members from social care, criminal justice and healthcare and be led by the Home Office.

## About the authors

**Stephen Attard** is a consultant forensic psychiatrist at Central and North West London NHS Foundation Trust, London, UK. **Solange Valdez-Symonds** is Supervising Solicitor and CEO at the Project for the Registration of Children as British Citizens, London, UK. **Steve Valdez-Symonds** is a solicitor and Refugee and Migrant Rights Programme Director at Amnesty International UK, London, UK. **Andrew Iles** is a consultant forensic psychiatrist at Surrey and Borders NHS Foundation Trust, Leatherhead, UK. **Frances Maclennan** is a consultant clinical psychologist at Central and North West London NHS Foundation Trust, London, UK.
